# Evaluating the Use and Feasibility of Indocyanine Green (ICG) as a Beacon of Precision in Sentinel Node Biopsy for Breast Cancer from an Oncoplastic Practice in India

**DOI:** 10.3390/cancers18061042

**Published:** 2026-03-23

**Authors:** Chaitanyanand B. Koppiker, Rupa Mishra, Vaibhav Jain, Sneha Bhandari, Namrata Athavale, Nutan Jumle, Chetan Deshmukh, Beenu Varghese, Upendra Dhar, Anushree Vartak, Pallavi Daphale, Laleh Busheri, Vishesha Lulla, Sneha Joshi

**Affiliations:** 1 Prashanti Cancer Care Mission (PCCM), Pune 411048, India; 2 Centre for Translational Cancer Research (CTCR), A Joint Initiative of IISER-PCCM, Pune 411048, India; rupamishra@prashanticancercare.org (R.M.); cda1@prashanticancercare.org (V.J.); cdc4@prashanticancercare.org (S.B.); namrata.athavale@prashanticancercare.org (N.A.);; 3 Jehangir Hospital, Pune 411001, India; 4 Orchids Breast Health Centre, in Association with PCCM and Jehangir Hospital, Pune 411001, India; 5 International School of Oncoplastic Surgery, Prashanti Cancer Care Mission, Pune 411048, India

**Keywords:** sentinel lymph node biopsy (SLNB), indocyanine green (ICG), breast cancer, axillary staging, oncoplastic surgery, low-resource settings

## Abstract

**Background:** Accurate axillary staging is vital in breast cancer. While dual tracers (Tc-99m + methylene blue dye) are standard for sentinel lymph node biopsy (SLNB), indocyanine green (ICG) offers a cost-effective, safe alternative, especially where nuclear medicine access is limited. Despite growing global use, data from low- and middle-income countries (LMICs) remain scarce. This study presents India’s largest cohort using ICG in SLNB. **Methods:** We analyzed data from 678 breast cancer patients (2013–2023), of whom 609 underwent SLNB. For analysis, patients were grouped into: isotope + blue dye (control), ICG + blue dye (study group), and ICG alone. False-negative rate (FNR) was evaluated in cases where SLNB was followed by axillary lymph node dissection (ALND). All other outcomes were assessed across the SLNB cohort. **Results:** In upfront surgery, the study group had an identification rate (IR) of 95.6%, an FNR of 5%, and a median node yield of four, compared to the control group (IR 94.1%, FNR 0%, median of three). Post-neoadjuvant systemic therapy (NAST), the study group outperformed the control (IR 92% vs. 88.2%; both FNR 10%), with higher node yield (three vs. two). From 2021, ICG alone showed 100% IR, 0% FNR (upfront), and 95.6% IR (post-NACT), with high median node retrieval. Overall recurrence was 7.8%; loco-regional recurrence was 3.09%. **Conclusions:** ICG offers high efficacy, safety, and feasibility as a sole tracer, especially in LMICs. Its integration into SLNB and oncoplastic workflows supports its broader adoption as a practical alternative to radioisotopes in breast cancer surgery.

## Simple Summary

Accurate staging of the axillary lymph nodes is essential for guiding treatment in breast cancer. Sentinel lymph node biopsy (SLNB) is the standard method for evaluating lymph node involvement and traditionally uses radioactive tracers combined with blue dye. However, this approach requires access to nuclear medicine facilities, which are often limited in many low- and middle-income countries (LMICs). In this study, we analyzed outcomes from 678 breast cancer patients treated at an oncoplastic breast surgery unit in India over a 10-year period, representing the largest reported Indian cohort evaluating indocyanine green (ICG) for SLNB. Our findings show that ICG demonstrated identification rates and accuracy comparable to or better than conventional tracers, with excellent node retrieval and very low false-negative rates, both in upfront surgery and after neoadjuvant therapy. When used alone, ICG achieved a 100% identification rate in upfront cases. Overall oncological outcomes, including recurrence rates, were favorable. In addition to being effective, ICG simplifies surgical logistics because it does not require radioactive tracers, making it particularly suitable for resource-limited settings. These results support ICG as a safe, practical, and scalable alternative for sentinel node mapping in breast cancer surgery.

## 1. Introduction

Breast cancer is the leading cause of cancer-related mortality among women worldwide. In 2022, an estimated 2.3 million women were diagnosed with breast cancer, resulting in approximately 670,000 deaths. Globally, the age-standardized incidence rate is estimated at 48 per 100,000 women, ranging from fewer than 30 per 100,000 in sub-Saharan Africa to more than 70 per 100,000 in Western Europe and North America. Although historically considered a disease of high-income countries, over half of new breast cancer cases and nearly two-thirds of related deaths in 2020 occurred in low- and middle-income countries (LMICs) [1]. In India, breast cancer continues to be the most common cancer, accounting for 28.8% of all female cancers, with an estimated 216,108 cases by 2022 [2]. Between 1990 and 2016, the age-standardized incidence rate of female breast cancer in India exhibited a significant increase of 39.1%. This upward trend was consistently observed across all states in the country over the past 26 years [3].

Like any other type of cancer, prognosis is largely related to the stage of the disease [4]. Axillary lymph nodes play a pivotal role in the metastatic spread of breast cancer, making axillary staging essential for assessing disease status and prognosis. Accurate evaluation of axillary involvement is crucial for guiding treatment decisions and optimizing patient outcomes [5].

For patients with clinically node-negative (cN0) breast cancer undergoing upfront surgery, the less invasive sentinel lymph node biopsy (SLNB) has largely replaced complete axillary lymph node dissection (ALND) as the gold standard for regional axillary staging due to the significant morbidity associated with ALND [6]. Results of the American College of Surgeons Oncology Group Z0011 (ACOSOG Z0011) randomized clinical trial demonstrated no benefit to performing ALND in comparison to not performing ALND, even in cases where up to two sentinel nodes are positive [7]. These results were further substantiated by other randomized controlled studies [8,9,10]. The European Society for Medical Oncology (ESMO) clinical practice guidelines recommend SLNB as the standard of care for patients with early breast cancer [11]. In line with this recommendation, the National Comprehensive Cancer Network (NCCN) guidelines specify that patients with cT1-2N0 breast cancer and two or fewer positive SLNs who are planned for radiation therapy, regardless of the type of breast surgery, should omit ALND. Additionally, in cT1-2N0 patients with two or fewer positive SLNs, the Japanese Breast Cancer Treatment Guidelines advise not performing ALND; however, this suggestion is restricted to patients having breast-conserving surgery followed by radiation therapy [12].

SLN mapping and localization technologies have evolved over time since their initial description in 1993 [13,14]. SLN biopsy in breast cancer patients was first done by injecting isosulfan (or methylene) blue (blue dye) into the tumor site [13]. After radioisotopes were developed as tracers and gamma probes, it became easier to identify the SLN [15]. Krag et al. were able to identify sentinel nodes in 18 (82%) of 22 patients using a radioisotope and gamma probe [16]. Morton et al. from the John Wayne Cancer Institute initially introduced the idea of dual mapping (using a dye and a radioisotope) in 1992, when they employed cutaneous lymphoscintigraphy before SLNB in melanoma [15]. Subsequently, dual mapping in SLNB was employed and proven to improve sensitivity and detection rate in patients with breast cancer [17,18]. In the NSABP-B32 trial evaluating the technical success and accuracy of SLN resection plus ALND versus SLN resection alone, the SLN detection rate using a combination of blue dye and the radioisotope method was 97.2% [19]. Despite the satisfactory detection and accuracy rates achieved by combining dye and isotope techniques for SLN identification, logistical challenges persist [20,21]. These include stringent safety protocols for handling and disposing of radioisotopes, staff training requirements, and regulatory hurdles imposed by local authorities. Moreover, access to radioisotopes is limited, particularly in developing countries [20,21]. A survey conducted in India during a national breast cancer congress reported that only 68.5% of surgeons offered SLNB for axillary staging in node-negative early breast cancer, primarily due to limited radioisotope availability and inadequate training [22]. Conversely, relying solely on blue dye is associated with drawbacks such as lower identification rates, risk of allergic reactions, the necessity for cumulative surgical experience, and the inability to preoperatively locate lymph node sites [20,21]. This scenario underscored the need for developing alternative methods that can match the detection and accuracy of existing techniques while mitigating their limitations [20,21].

Indocyanine green (ICG) has been utilized in clinical practice since the mid-1950s [23]. Motomura et al. first reported the results of SLNB using ICG dye alone [24,25]. Subsequently, the group demonstrated that the combination of ICG and radioisotopes is superior to dye alone for SLNB [26]. Over the years, there has been an increasing trend in the use of ICG in surgery [27]. Its near-infrared fluorescence window (700–900 nm) offers advantages, such as minimal autofluorescence of the surgical field and increased penetration of the fluorescent light through the tissue [28]. While the depth of penetration of ICG is greater than that of blue dye, it must be noted that radiotracers have far superior penetration, albeit with the associated risk of exposure [29].

The aim of the current study was to retrospectively evaluate the feasibility, efficacy, and safety of using ICG as a single tracer for SLNB in breast cancer patients in a low-resource setting in a low- and middle-income country (LMIC). Comparison of this technique with conventional methods is also performed.

## 2. Methods

### 2.1. Study Design and Setting

This was a retrospective observational study of patients from 2013 to 2023 treated at our single onco-surgeon unit. The study included breast cancer patients who were candidates for axillary management. The 678 patients included in the study were diagnosed with malignant breast tumors, underwent a thorough evaluation, and were found to be candidates for axillary management. Data were retrieved from a comprehensive institutional database of breast cancer cases treated during this period. The study was conducted in accordance with the Declaration of Helsinki, and approval was obtained from the Independent Ethics Committee. Patient consent for the use of clinical data was obtained in line with institutional policies, ensuring confidentiality and adherence to ethical standards for data protection.

### 2.2. Patient Selection Criteria

**SLNB Algorithm—Prashanti Cancer Care Mission (PCCM) Breast Cancer Unit**
**Step 1: Initial Axillary Evaluation**

**SLNB Algorithm:** As part of the SLNB algorithm validation, three representative surgical cases and a short surgical video demonstrating the intraoperative “Beacon Sign” are included in the Supplementary Material.

**a.**  **Clinical Evaluation**

- Routine axillary palpation by the breast surgeon to check for palpable nodes.

**b.**  **Radiological Evaluation (2013–2023)**

● **Radiological evaluation** via **ultrasound of the axilla** in all patients.
● If suspicious nodes are identified → considered for ALND or neoadjuvant systemic therapy (NAST) based on clinical context.
  ○ **Nodal staging is assigned based on combined clinical and imaging findings**
  ○ **Between 2013 and 2023, only radiological appearance was used to assess nodal status.**
▪ **cN0:** No evidence of cancer in the lymph nodes on clinical exam and imaging.
▪ **cN1:** Minimally suspicious or involvement of 1–3 axillary lymph nodes that are **not fixed or matted**.
▪ **cN2:** Involvement of **fixed/matted** axillary lymph nodes or internal mammary nodes.
▪ **cN3:** Involvement necessarily of Level III axillary lymph nodes or clinically evident nodes or ipsilateral supraclavicular lymph node(s).

**Step 2: Determine Clinical Node Status (cN)**

● **cN0 (node negative).**
  ○ **Normal axillary exam.**
  ○ **Normal or benign-looking lymph nodes on imaging.**

(oval, thin cortex, preserved hilum)

⋅ **Proceed to SLNB.**
● **cN1 (minimally suspicious nodes)** (e.g., indeterminate morphology on ultrasound, i.e., mild cortical thickening).
  ○ **Treated as SLNB eligible unless strongly suggestive of malignancy.**
    ⋅ **Proceed to SLNB.**
● **cN1 (confirmed clinically or radiologically with few abnormal nodes).**

→ **Proceed to SLNB and confirm, then proceed with** ALND or NAST, followed by re-staging, which represents an aggressive form of disease.
  ● **cN2–cN3 (clinically or radiologically abnormal nodes, fixed/matted or high-volume disease).**

→ ALND or NAST followed by re-staging.

**Step 3: Post-NAST Reassessment**

● **All patients who receive NAST undergo repeat axillary ultrasound.**
● If nodes appear normal post-NAST (ycN0):
⋅ **SLNB performed.**
● If nodes remain abnormal (ycN1) → **ALND performed.**

**Step 4: Intraoperative SLNB Management**

- SLNB is performed in all **cN0 and downstaged cN_+_** → ycN0
- SLNs are assessed intraoperatively (frozen).
  ○ **If >2 SLNs positive: → ALND performed.**
  ○ **If 1–2 SLNs positive, postmenopausal, early-stage: → Z0011 criteria applied.**
  ○ **If SLNs negative: No further axillary dissection.**

**Please note: Ultrasound-guided fine needle aspiration cytology (FNAC; added 2023 onwards).**

- FNAC performed for **radiologically abnormal/suspicious nodes.**
  ○ **FNAC-positive** → **ALND or NAST.**
  ○ **FNAC-negative** → **SLNB eligible.**
  ○ If not accessible for FNAC, surgical decision made based on radiological appearance, like before.
  ○ **SLNB Techniques and Tracers Used.**

Patients were categorized based on the type of tracer used for SLNB. These included radioactive colloid (technetium-99 m), blue dye (methylene blue), and ICG used in conjunction with fluorescence imaging for real-time SLN localization. SLNB was performed following standard institutional protocols, which involved marking SLN using the chosen tracer(s) prior to surgery. In cases where ICG was used, a fluorescence imaging system (SPY-PHI, Stryker, Kalamazoo, MI, USA) was employed to visualize the SLN in real-time during surgery. The identification of SLNs was confirmed by visual inspection, and additional SLNs were excised if intraoperative findings indicated further sentinel node involvement. The surgical approach adhered to established guidelines to ensure consistency and reliability in the procedure. All resected SLNs were sent for frozen section evaluation during surgery. After surgery, routine paraffin section pathological examination of SLNs is a standard practice.

### 2.3. Outcomes Assessed

The primary outcomes of the study were the identification rate (IR) and the false-negative rate (FNR). IR was defined as the proportion of patients who had successful localization of the SLN during surgery, while the FNR was determined by calculating the percentage of SLNB-negative cases (those with no sentinel node involvement) that were later found to have metastatic involvement upon completion of ALND. These outcomes provided insight into the diagnostic accuracy of SLNB across different tracer techniques. Secondary outcomes included oncological outcomes, quality of life (QoL) assessments, safety and adverse events. Oncological outcomes were assessed by monitoring the incidence of local recurrence and distant metastasis during the follow-up period. QoL was evaluated using standardized instruments to assess the impact of SLNB and axillary interventions on patient well-being, focusing on factors such as lymphedema and functional arm recovery, including range of motion and arm strength. The safety of the procedure was assessed by recording any complications associated with SLNB, including infection, seroma, hematoma, or allergic reactions to the tracers used. Additionally, the study evaluated the feasibility of implementing different SLNB techniques in low-resource settings.

### 2.4. Statistical Analysis

Data were analyzed using Power BI (Version: 2). Descriptive statistics were employed to summarize patient demographics, tracer groups, and outcome measures. Continuous variables were reported as means with standard deviations (SD) or medians with interquartile ranges (IQR), depending on the distribution of the data. Categorical variables were summarized as frequencies and percentages. IR and FNR were calculated for each tracer group. Statistical significance was assessed using the conventional threshold of *p* < 0.05, which is considered an acceptable balance between control of Type I error (false-positive findings) and statistical power in the vast majority of biomedical research. Subgroup analyses were performed to assess the impact of factors such as age, tumor size, and type of tracer on primary and secondary outcomes. Kaplan–Meier survival analysis was used to evaluate time to local recurrence or distant metastasis.

### 2.5. Surgical Methods

This section outlines the standardized surgical methodology employed for SLNB in breast cancer surgeries at our center, with particular emphasis on the technique used when integrating ICG as a lymphatic tracer. This study was conducted in a single oncoplastic unit led by one primary oncoplastic surgeon, supported by trained assistant surgeons. All surgical procedures, including SLNB procedures, whether using radioisotopes with blue dye, ICG with blue dye, or ICG alone, were directly performed by the same lead surgeon following standardized institutional protocols.

### 2.6. The “Beacon” Sign

Once ICG is injected, lymphatic flow is visualized in real time using a near-infrared camera system. During axillary dissection, the surgeon follows the fluorescent signal. A distinct phenomenon, coined “the Beacon” by our team, guides this process. The Beacon refers to the bright, focused flash of ICG fluorescence that appears during dissection, signaling the precise location of the sentinel lymph node. It functions as a visual guide—akin to a lighthouse—enabling targeted and limited dissection (Supplementary Video S2).

### 2.7. ICG Preparation and Injection Technique and Intraoperative Considerations

In our protocol, 25 mg of ICG is diluted in 10 mL of sterile water to yield a concentration of 2.5 mg/mL. From this, 0.05 mL is injected intradermally in peri-areolar location. In some settings, ICG could also be injected either intradermally or subcutaneously (1 mL) in periareolar or peritumoral sites, respectively, depending on the tumor location and quadrant. Our approach is towards maximizing breast conservation and the practice uses aid of oncoplastic techniques in breast conservation. Intra-dermal peri-areolar ICG injection, often interfered with intraoperative visualization during techniques like therapeutic mammoplasty due to widespread dispersion of dye into breast tissue. Based on this, the injection technique was refined. The current preferred approach involves injecting ICG 2–4 cm superolaterally from the areolar complex or at the proposed site of nipple repositioning in oncoplastic procedures. This allows the dye to flow fast and effectively into the axillary lymphatics while preserving surgical planes and visibility. In India, specialized breast centers handle many post-lumpectomy early breast cancer cases with cN0 axilla. In cases with periareolar scars, a poor SLNB identification rate is a challenge, which can be mitigated by using an ICG injection site in the upper outer quadrant or lateral to the location of the excision cavity. In cases where the tumor is located closer to the axilla, or there is a suspicion of blocked lymphatic drainage (e.g., post-neoadjuvant chemotherapy [NACT] fibrosis), an additional injection at the peri-tumor site may be used. Volume of injection is one of the factors that needs to be considered to choose between these two options, along with the time taken between injection and the removal of SLN, where a longer time (15–20 min) may lead to diffusion. Gentle breast massage accelerates dispersion of the dye and should be used with caution to achieve optimal time from injection to SLN assessment, as well as to avoid staining of the field. SLNs typically fluoresce within 5–7 min, visualized using a handheld multispectral near-infrared camera (805 nm).

In the overlay mode, which allows fluorescence visualization and white light imaging simultaneously, meticulous dissection of lymphatics can be performed to avoid spillage due to ambient light immunity. It also facilitates visualization of blue dye if using a dual-dye method. Color-segmented fluorescence mode presents increasing intensity of fluorescence in the scale of gray to red and can help identify/distinguish small-sized SLNs embedded in fibrofatty axillary tissue.

Oncoplastic breast surgery involves varied incision patterns and often avoids standard axillary incisions to preserve aesthetic outcomes. In such cases, SLNB is performed through remote approaches—commonly via lateral pillar incisions in therapeutic mammoplasties or through flap incisions in latissimus dorsi (LD) or lateral thoracic artery perforator (LTAP) reconstructions. The *Beacon* remains critical, as fluorescence can be visualized even through several millimeters of adipose tissue, guiding dissection from a distance. This is particularly advantageous in avoiding additional auxiliary incisions. Dissection is directed along the lateral border of the pectoralis or the anterior border of the latissimus muscles, entering the axilla only at necessary points. Notably, the SLN often lies medial to the lateral thoracic artery, within a triangle bounded by the artery and the intercostobrachial nerve. Removal of the SLN reveals these landmarks, further aiding safe dissection.

- Node Count: The goal is to retrieve at least 3 SLNs to ensure accurate staging. In post-NAST settings, up to 5 nodes are accepted to account for altered lymphatic drainage and potential fibrosis. However, variations in patient physiology, such as richer lymphatic networks or clustered sentinel nodes, may lead to higher node pickup in some cases, which is considered acceptable as long as dissection remains targeted and anatomical planes are preserved. Landmark trials such as SENTINA have shown that SLNB after NAST achieves acceptable FNRs (<10%) when three or more SLNs are retrieved, with FNRs rising with fewer nodes removed [30,31,32]. These data underpin the recommendation to aim for ≥3 SLNs in the post-NAST setting to optimize diagnostic accuracy.
- Incision Length: A slightly wider incision (2–2.5 cm) is preferred over multiple small entries to allow anatomical structure visualization and minimize trauma.
- Dye Diffusion Time: In post-NAST cases, lymphatic mapping with ICG may require additional time due to fibrotic changes. However, the dye consistently reaches axillary nodes, and fluorescence facilitates node identification even under challenging conditions.
- Imaging: The ICG signal is detectable from a distance, unlike blue dye, allowing for non-invasive navigation toward nodes.
- Future Directions: Although not routinely performed, preserving secondary lymphatics and mapping through remote incisions holds potential for advanced lymphatic-sparing approaches.

## 3. Results

### 3.1. Overview of the Study Cohort

**Figure 1:**
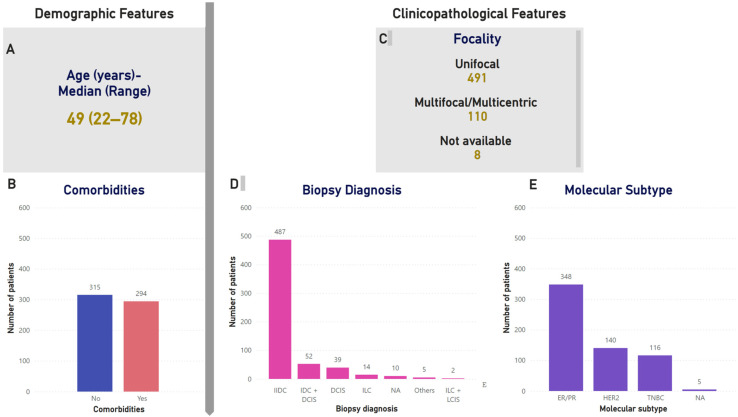
Demographic and clinical characteristics of the cohort. (**A**) Median age at diagnosis was 49 years. (**B**) Distribution of comorbidities in the cohort. (**C**) Focal distribution in the cohort. (**D**) Distribution of biopsy diagnosis in the cohort. (**E**) Molecular subtype distribution.

The overview of the surgical cohort is presented in Table 1. Demographic and clinical characteristics of the study participants are presented in Figure 1 and Table 2 and Table 3. This retrospective study reports on a cohort of 678 breast cancer patients, of whom 609 underwent SLNB alone or SLNB followed by ALND between 2013 and 2023. As this was a retrospective analysis over a decade, complete data were not available for all variables for every patient; therefore, the totals for certain parameters may not always sum to 609. The median age at diagnosis was 49 years (range 22–78 years). Additionally, comorbidities such as diabetes and hypertension were prevalent in 48% (294/609) of the patients.

Of the 609 cases, 588 patients had unilateral malignant disease, while 20 patients presented with bilateral malignancies. Unifocal malignant disease was observed in 491 patients, whereas 110 patients exhibited multifocal or multicentric disease.

Around 67% of patients were diagnosed between stages IA and IIA, with 70% of the total SLNB cohort presenting as clinically node-negative. The detailed clinicopathological characteristics of the cohort are comprehensively summarized in Table 2 and Table 3.

**Figure 2:**
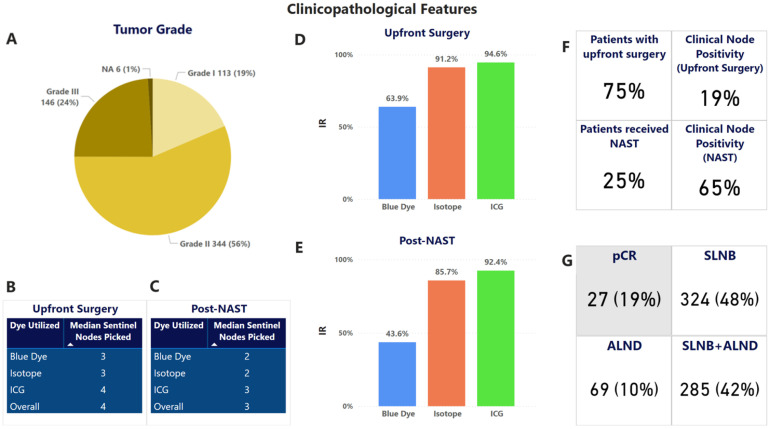
Clinicopathological features and oncological outcomes of the cohort. (**A**) Tumor grade. (**B**) Distribution of tracer utilization and node pickup in upfront. (**C**) post-NAST settings. (**D**) Identification rate based on the tracer used in the upfront surgery setting. (**E**) Identification rate based on the tracer used in the post-NAST setting. (**F**) Clinical node positivity in upfront and post-NAST settings. (**G**) pCR rates and distribution of approach to axilla management in the cohort.

Subtype distribution for breast cancer patients, as shown in Table 2 and Figure 1, was ER/PR (estrogen/progesterone receptor) 57% (348/609), HER2+ (human epidermal growth factor receptor 2) enriched 23% (140/609), and TNBC (triple-negative breast cancer) 19% (116/609). Of the 609 breast cancer patients, 147 (24%) received NAST, with a pathological complete response (pCR) in 19% (27/147) of cases. Figure 2A summarizes the tumor grade distribution of the breast cancer patients in the cohort.

### 3.2. SLN Detection Efficiency in Upfront Surgery and Post-NAST Patients

**Overall Cohort:** Of the cohort of 678 breast cancer patients, 609 underwent SLNB, while the remaining patients proceeded directly to ALND. Among the 609 patients who underwent SLNB, 343 did not have positive nodes on SLNB detection. However, ALND was performed in 21 of these patients, revealing occult nodal metastases in three cases. SLNB was positive in 239 patients. Of these, 165 underwent ALND, while 73 patients met the ACOSOG Z0011 criteria (with only 1–2 positive SLNs) and were managed with adjuvant therapy instead of ALND.

Of 609 cases, 457 (75%) underwent upfront surgery, while 147 (25%) received NAST. The median number of SLNs retrieved was four in upfront surgery patients and three in post-NAST patients. SLN detection rates varied across different tracers. ICG demonstrated the highest efficiency in both groups, with a 94.6% detection rate and a median retrieval of four nodes in upfront surgery patients and 92.4% detection efficiency with a median of three nodes in post-NAST patients. The isotope tracer followed, with 91.2% detection and a median of three nodes in upfront surgery patients, compared to 85.7% detection with a median of two nodes post-NAST. Blue dye exhibited the lowest detection efficiency, with 63.9% detection and a median of three nodes in upfront surgery patients, and 43.6% detection with a median of two nodes in post-NAST patients (Figure 2B–E).

**Analysis Sub-Cohort:** In the upfront surgery setting, as presented in Table 4, the control group (isotope + blue dye) demonstrated an FNR of 0% (0/33), an IR of 94.1%, and a median sentinel node pickup of three. In comparison, the study group (ICG + blue dye) showed an FNR of 5% (1/18), a marginally better IR of 95.6%, and a higher median node retrieval of four. In the post-NAST setting, the control group had an FNR of 10% (3/30), an IR of 88.2%, and a median node pickup of two. The study group performed better in this context, also with an FNR of 10% (2/20), but with a higher IR of 91.2% and a median node pickup of three, suggesting improved detection efficiency with the addition of ICG. From 2021 onwards, we gradually transitioned to ICG alone as the preferred tracer. This group showed the highest overall IR of 100% with 0% FNR in the upfront setting and a median of four nodes retrieved. In the post-NACT setting, ICG alone maintained a strong performance with an IR of 95.6% and a median node pickup of three.

### 3.3. Surgical Outcomes

#### 3.3.1. Surgical Margins

**Figure 3:**
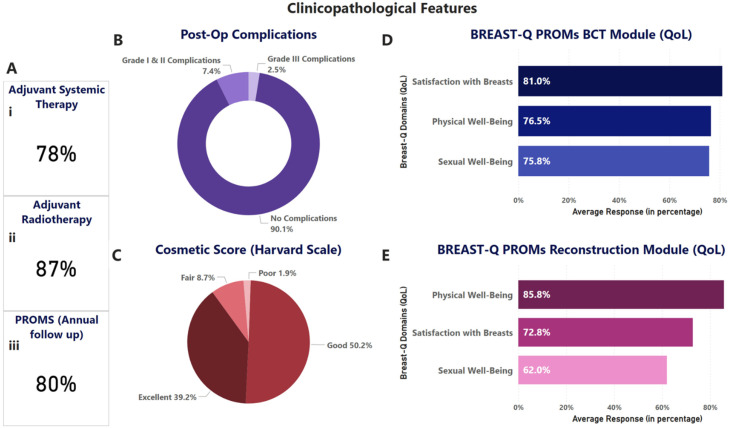
Clinicopathological features and oncological outcomes. (**A**) Adjuvant therapy and patient-reported outcome measures (PROMs) response rate. (**B**) Post-operative complication rate in the cohort. (**C**) Cosmetic scores post-surgery in the cohort. (**D**) PROMs outcomes in the BCT module. (**E**) PROMs outcomes as per the Reconstruction module.

Among the 609 patients who underwent SLNB with or without ALND, 486 (80%) underwent breast conservation with or without the aid of oncoplasty, and 123 (20%) underwent mastectomy with or without reconstruction. Through the incorporation of intraoperative frozen section pathology during the surgical procedure, clear margins were achieved in all breast cancer cases. Details of the surgical outcomes are given in Figure 2F,G and Figure 3.

#### 3.3.2. Post-Operative Complications

Post-operative complications were classified based on grades as per the Clavien–Dindo classification adapted for breast cancer^25^. Complications were observed in 9.9% of cases, similar to observations reported in earlier literature. All complications were treated conservatively in the outpatient setting. In general, we observed a low rate of complications immediately post-surgery, with a major proportion being Grade I/II complications (Figure 3B).

### 3.4. Adjuvant Radiotherapy (RT)

Overall, 87% of the cohort underwent RT as clinically indicated. Among those who received RT, 46.5% of patients developed minor complications post radiation (Grade I–II reactions), while only 0.9% of patients developed Grade III reactions.

### 3.5. Survival Outcomes

Survival data were available for 507 patients from the total cohort of 609. At a median follow-up of 48 months, the overall survival (OS) was 98.38%, and the disease-free survival (DFS) was 91.43%. In the subset of patients where ICG was used as a dual or single tracer (n = 356), the median follow-up was 38 months, with OS of 98.79%, while DFS was 93.61%. The overall axillary recurrence rate across the cohort was 0.4%, with two axillary recurrences noted. The overall mortality observed was 1% (n = 6). Axillary recurrence-free survival at median follow-up was observed to be 99.78%. The overall recurrence rate was 7.8%, while local recurrence was observed to be 3.09% in the cohort.

**Figure 4:**
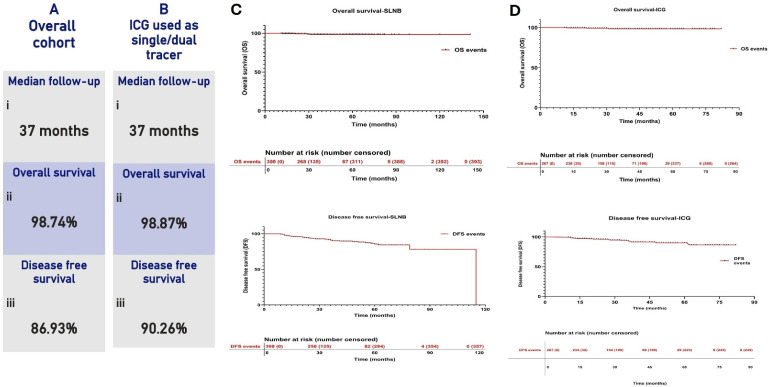
(**A**) Follow-up and survival statistics in the overall SLNB cohort. (**B**) Follow-up and survival statistics in a cohort wherein ICG was used either as a single or dual tracer. (**C**) OS and DFS in SLNB cohort. (**D**) OS and DFS in the ICG cohort. Number at risk is presented below the graph.

Kaplan–Meier survival curves for OS and DFS in the SLNB cohort and ICG cohort are depicted in Figure 4.

### 3.6. Cosmetic Score Analysis

Cosmetic scores were analyzed for all surgeries by surgeons within 3–6 months post-surgery. Cosmetic scores were majorly good to excellent across all surgery types. Figure 3C shows the cosmetic scores as reported by the surgeons. Satisfaction with breasts in the Breast-Q PROMs analysis also showed an average score of 85%.

### 3.7. PROMs

BREAST-Q-based PROMs data were collected from the study participants after a minimum period of 12 months post-surgery using the BREAST-Q questionnaires. Out of 609 breast cancer patients, 480 (80.0%) responded to the questionnaire. The analysis demonstrated high post-surgical QoL scores across all breast surgery procedures. Patients undergoing breast conservation therapy (BCT) reported scores of 72.45% for sexual well-being, 81.0% for satisfaction with breasts, and 74.8% for physical well-being, suggesting a balanced aesthetic and functional outcome. Patients who underwent whole breast reconstruction post mastectomy reported 76.6% on physical well-being, satisfaction with breasts at 72.6%, and sexual well-being at 66.6%. Overall, PROMs data reflect favorable QoL outcomes, particularly in the breast conservation group (Figure 3D,E).

### 3.8. Trends in the Utilization of SLNB Tracers at Our Center

**Figure 5:**
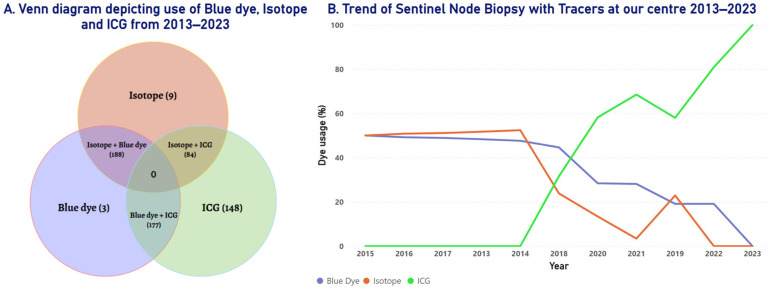
Trend of SLNB tracers used at the center between 2013 and 2023. (**A**) Depicts the usage of blue dye, ICG, and isotopes and their overlap. (**B**) Depicts the trend in tracer usage at the center between 2013 and 2023.

Figure 5 illustrates utilization patterns of SLNB tracers—blue dye, isotope, and ICG—over a 10-year period from 2013 to 2023. A marked shift in SLNB tracer utilization was observed at our center over the last decade. Initially, blue dye and isotope were the predominant tracers used, with consistent application through 2018. However, following the introduction of ICG in 2018, its use rose sharply. By 2018, ICG had become the most frequently used tracer, and by 2020, it was used in over 90% of cases as a single tracer. Concurrently, the use of blue dye and isotopes declined significantly, becoming nearly obsolete by 2021.

## 4. Discussion

The findings of our study align with existing evidence supporting the use of ICG in SLNB and further strengthen its role as a reliable single tracer, particularly in low-resource settings, while uniquely contributing comprehensive data on oncological safety, cosmetic outcomes, PROMs, and survival—validating both our clinical algorithm and its broader applicability. In our cohort, of the 609 patients undergoing SLNB, the majority were accurately staged, with low FNRs and appropriate application of Z0011 criteria. ICG consistently outperformed isotope and blue dye in both upfront and post-NAST settings, achieving the highest IRs—94.6% in upfront surgery versus 91.2% for isotope and 63.9% for blue dye, and 92.4% post-NAST compared to 85.7% and 43.6%, respectively. Median node retrieval was also highest with ICG (four nodes upfront, three post-NAST) versus isotope (three and two) and blue dye (three and two). In our analysis subgroup, ICG + blue dye achieved an IR of 95.6% versus 94.1% for the control group and a higher median node count (four vs. three). Transitioning to ICG alone from 2021 yielded a 100% IR and 0% FNR in upfront cases. Clinically, this translated into accurate staging, 98.38% OS, and 91.43% DFS at a median follow-up of 38 months. Oncoplastic procedures showed excellent margin control (100% clear margins) and low complication rates (9.9%, mostly Grade I–II). A notable outcome in our study is the 0% rate of positive margins, achieved through the routine use of intraoperative frozen section analysis. While this approach has drawn criticism due to concerns over its reliability and resource intensity, our experience demonstrates its efficacy [33,34,35]. We have a dedicated, experienced pathologist present during surgeries who performs detailed real-time assessments, enabling immediate corrective action when needed. This approach minimizes the need for re-excision and second surgeries—an essential factor in our LMIC setting, where patients often face significant financial barriers to multiple hospital visits. Moreover, our low local recurrence rate further validates the effectiveness of this protocol. It is important to acknowledge that several studies have reported inherent limitations of intraoperative frozen section analysis in margin assessment for invasive carcinoma, including suboptimal sensitivity and the risk of false-negative results due to sampling and interpretive challenges; however, these limitations are highly operator- and system-dependent and can be mitigated in centers with dedicated breast pathology expertise and standardized protocols [34,36,37]. In a previous publication [38], blinded re-evaluation of pathology slides by an independent international pathologist confirmed the accuracy of our negative margin reporting, reinforcing the robustness of our surgical and pathology systems. Also, in our setting, patient-reported outcomes were favorable, with an average Breast-Q satisfaction score of 85% and good-to-excellent cosmetic ratings across surgeries. These results highlight the feasibility of integrating ICG into both standard and advanced oncoplastic workflows, particularly in low-resource settings, balancing oncological safety with superior functional and aesthetic outcomes for patients. The representative cases, along with surgical videos, are added to the Supplementary Material. These materials highlight the utility of ICG in enabling clear visualization of SLNs in simple conservation surgeries (Supplementary Case S1 and Supplementary Video S1) as well as in oncoplastic (Supplementary Case S2 and Supplementary Video S2) and post-NAST (Supplementary Case S3 and Supplementary Video S3) settings, guiding precise dissection from remote incisions, and enhancing surgical accuracy while minimizing axillary disruption. The bright fluorescence, visible even from a remote incision, guided precise and minimally invasive dissection. We illustrate this step in Supplementary Video S2 as the “Beacon Sign,” which enhances surgical accuracy while preserving tissue planes and minimizing axillary disruption—reinforcing the practical advantages of ICG-guided SLNB in routine and oncoplastic surgical settings.

The use of ICG fluorescence in SLNB has emerged as a promising alternative to traditional techniques, particularly in breast cancer management. ICG-guided SLNB has demonstrated high IR and low FNR, comparable to the gold-standard combination of radioisotope and blue dye. While single-tracer SLNB has been done before using the existing methods, the inherent disadvantages related to these methods have given way to the exploration of ICG as a single tracer for SLNB.

In upfront surgery, multiple studies confirm the high diagnostic accuracy of ICG for SLNB, including a meta-analysis of 19 studies, which reported an IR of up to 98% with ICG alone, outperforming even dual-agent techniques [39]. Other meta-analyses similarly support its efficacy [40,41]. The INFLUENCE trial and multiple other studies endorse ICG as a reliable single- or dual-agent tracer for routine SLNB [42,43,44,45,46,47]. Aside from upfront surgery, several studies also support the feasibility and safety of ICG for SLNB in breast cancer patients post-NAST, reporting IRs between 91.5% and 97.4%, with no complications [48,49]. A study comparing tracers post-NACT found ICG to have the highest nodal detection (93.2%) over blue dye (81.8%) and radioisotope (53.9%). Combining ICG and blue dye improved IR to 96.7%, with FNRs dropping below 10% when all three tracers were used [50].

Beyond diagnostic accuracy, emerging evidence highlights the prognostic significance of SLNB in the post-neoadjuvant setting. A recent study evaluating single-tracer SLNB after NAST in 322 patients demonstrated that SLN status retained strong prognostic value, with significant associations between nodal response and both DFS and OS [51]. Importantly, patients with negative SLNs after NAST showed excellent oncologic outcomes despite omission of ALND, supporting axillary de-escalation strategies when SLNB is appropriately performed [51]. These findings reinforce that single-tracer SLNB is not merely a technically acceptable alternative but a clinically and prognostically meaningful tool in modern breast cancer management.

Economic evaluations also strongly support the cost-effectiveness of ICG in SLNB. A study on 291 patients found ICG costs to be only 21.9% of those for 99Tc, with added logistical savings [52]. Another study of 295 breast reconstruction cases showed that routine ICG angiography reduced ischemic complications from 14.9% to 8.8%, gaining 1.77 quality-adjusted life-years (QALY) at AU$656 per QALY [53]. A multidisciplinary assessment also reported an 18% cost reduction per care pathway with ICG, alongside improved workflows and patient experience [54]. These findings are especially relevant in our LMIC setting, where affordability is key to clinical adoption.

ICG fluorescence imaging is increasingly valuable in oncoplastic breast surgery for assessing tissue perfusion and reducing complications. A 2024 study in nipple-sparing mastectomy (NSM) with reconstruction reported that ICG angiography predicted perfusion-related complications (PRC) with 84.6% sensitivity and 76.9% specificity, reducing PRC rates to 11.2% [55]. Real-time ICG visualization has also been shown to improve intraoperative decision-making by identifying ischemic zones during mastectomy flap creation [27]. Studies have shown that its use reduces flap necrosis and delayed wound healing, supporting its routine integration into oncoplastic workflows [56,57]. An umbrella review of 14 syntheses confirmed ICG’s role in minimizing complications and highlighted the need for standardized protocols [58]. The gBREAST-22 study further demonstrated ICG’s utility, reporting 71.4% sensitivity and 93.5% specificity in detecting intraoperative skin necrosis [59].

In the post-NAST breast conservation cohort, SLNB remains technically challenging due to chemotherapy-induced fibrosis, altered lymphatic drainage, and reduced nodal volume, all of which may compromise SLN identification. In our retrospective cohort, ICG-guided SLNB demonstrated reliable performance in this setting, with consistently high IRs and adequate SLN retrieval despite these challenges. Real-time fluorescence imaging enabled visualization of lymphatic channels and SLNs even in the presence of post-NAST tissue changes, facilitating targeted dissection with minimal axillary disruption. The median number of SLNs retrieved in post-NAST patients was three, meeting accepted oncological standards for representative sampling. These findings support the feasibility and safety of ICG-guided SLNB in patients undergoing breast conservation after NAST and reinforce its role in enabling axillary de-escalation without compromising staging accuracy.

Building on this evidence, our group is currently conducting a prospective study evaluating the role of intraoperative ICG fluorescence imaging in oncoplastic breast surgery, specifically in perforator flap vascularity assessment and real-time perfusion mapping. Preliminary findings suggest that ICG guidance facilitates precise pedicle assessment during therapeutic mammoplasty, helping surgeons verify flap viability before inset. This approach has been effective in reducing flap ischemia, decreasing rates of necrosis, and avoiding re-exploration or secondary surgeries. By enabling timely intraoperative adjustments, ICG imaging enhances surgical outcomes, improves patient safety, and significantly reduces reconstructive complications.

This study, while based on one of the largest single-center cohorts from India, has a few limitations. First, it is a single-institution experience and may reflect selection biases inherent to a high-volume oncoplastic referral center. Additionally, the study spans over 10 years, and the surgeon′s understanding and experience evolving over the period may act as a confounding factor influencing identification rates. Moreover, patients were not randomly assigned to receive ICG or radioisotope tracers. Tracer allocation was influenced by time period, tracer availability, and evolving institutional practice. These factors should therefore be considered when interpreting differences between tracers in this audit. Second, although the data were prospectively collected, the analysis was retrospective in nature, which may limit generalizability. Third, while ICG was used in various combinations and as a single agent, the shift to ICG alone occurred over time, and uniformity in tracer use across all cases was not maintained. Furthermore, changes in the ICG injection technique during the study period may have influenced the IR and FNR. Additionally, real-time documentation of time-to-node visualization and quantitative fluorescence intensity was not consistently recorded, which could have further strengthened comparisons across tracers. Lastly, while our center benefits from a dedicated intraoperative pathology team and specialized surgical expertise, replicating this model may appear challenging in other low-resource settings lacking similar infrastructure. However, we strongly recommend that such an algorithm be established where feasible, given its clear clinical and patient-centered benefits. Although we do not yet have exact financial comparisons between ICG, radioisotope, and blue dye methods, the logistical simplicity and observed outcomes make this protocol a practical and scalable option.

## 5. Conclusions

In conclusion, our study reinforces the clinical reliability, oncological safety, and patient-centered advantages of ICG as a single tracer for SLNB, particularly in resource-limited settings. With superior IRs, significant ease of use, excellent cosmetic outcomes, and high patient satisfaction, ICG not only streamlines axillary staging but also integrates seamlessly into oncoplastic workflows. Unlike radioisotopes, ICG eliminates the logistical burden of nuclear medicine coordination, avoids radiation exposure risks to patients and healthcare staff, and simplifies workflow without requiring specialized licensing or infrastructure. Its cost-effectiveness, ease of use, and safety profile further enhance its appeal, making it a practical and scalable solution for equitable, high-quality breast cancer care even in LMICs.

## Figures and Tables

**Table 1:** Total surgical cohort reported (2013–2023, n = 678).

Surgical Management Category	Total Number of Patients n = 678	Upfront Surgery n = 473	Post-NAST n = 200
SLNB Only	324	281	38
SLNB + ALND	285	176	109
ALND without SLNB	69	16	53

**Table 2:** Demographic and clinicopathological features of the cohort (n = 609).

Feature	Class	n	Percentage
Age (years)	Median (Range)	49 (22, 78)	
Comorbidities	Yes	294	48
	No	315	52
Tumor Grade	I	113	18.5%
	II	344	56.5%
	III	146	24%
	NA	6	-
Type of Tumor	IDC	487	80%
	IDC + DCIS	52	8.5%
	ILC	14	2.3%
	ILC + LCIS	2	0.33%
	DCIS	39	6.4%
	Others	5	0.8%
	NA	10	-

IDC= invasive ductal carcinoma; DCIS= ductal carcinoma in situ; ILC= invasive lobular carcinoma; LCIS= lobular carcinoma in situ; NA= not applicable.

**Table 3:** Clinicopathological features of the cohort in the upfront and post-NAST setting.

Feature	Class	Total: n = 609 (SLNB Yes)					
		Total n	% of Full Cohort	Upfront Surgery n = 457	Upfront Surgery 75%	Post NAST n = 147	Post-NAST 25%
Molecular Subtypes	ER/PR	348	57%	285	81%	63	16%
	HER2	140	23%	93	65%	45	33%
	TNBC	116	19%	76	65%	38	33%
	Not Available	5	-	-	-	-	-
Focality	Unifocal	491	81%	371	75%	118	24%
	MF/MC	110	22.3	82	75%	27	25%
	Not Available	8	-	-	-	-	-
Clinical Tumor (cT) n = 609	cTis/cT0	38	6.1%	34	89.5%	4	10.5%
	cT1	225	37%	189	84%	35	16%
	cT2	300	49%	198	66%	98	33%
	cT3	45	7.3%	35	77.8%	10	22.2%
	cT4	1	0.16%	1	100%	NA	NA
Clinical Node (cN) n = 609	cN0	427	70.1%	370	86%	52	12.2%
	cN1	121	20%	68	56.2%	53	43.8%
	cN2	56	9.1%	16	28.6%	40	71.4%
	cN3	5	0.8%	3	60%	2	40%
Clinical Node Positivity		370 of 457 (81%) were node negative and 87 of 457 (19%) were clinical node positive			95 of 147 (64.6%) were node positive and 52 of 147 (35.4%) were clinical node negative		
Clinical Tumor Stage n = 609	Stage 0	32	5.25%	29	90.6%	3	9.4%
	Stage IA	176	29%	159	90.3%	16	9.1%
	Stage IB	0	-	0	-	0	-
	Stage IIA	231	38%	182	78.8%	45	19.5%
	Stage IIB	100	16.4%	65	65%	35	35%
	Stage IIIA	64	10.5%	18	28.1%	46	71.9%
	Stage IIIB	1	0.16%	1	100%	-	-
	Stage IIIC	5	0.8%	3	60%	2	40%
	NA	-	-	-	-	-	-
Pathological Tumor Stage n = 609	Stage 0	50	8.2%	22	44%	28	56%
	Stage IA	121	19.8%	91	75%	29	25%
	Stage IB	1	0.16%	-	-	1	100%
	Stage IIA	221	36.3%	180	81.4%	37	16.7%
	Stage IIB	121	19.8%	101	83.5%	20	16.5%
	Stage IIIA	52	8.5%	37	70%	15	30%
	Stage IIIB	3	0.5%	2	67%	1	33%
	Stage IIIC	26	4.2%	13	50%	13	50%
	NA	14	-	-	-	--	-

ER/PR= estrogen/progesterone receptor; HER2= human epidermal growth factor receptor 2; TNBC= triple-negative breast cancer; MF/MC= multifocal/multicentric; NA= not applicable.

**Table 4:** Sub-cohort analysis comparing IR and FNR among the different tracers used for SLN identification.

	Sub-Cohort for Analysis	For IR n	IR (95% CI)	For FNR n	FNR (95% CI)
Upfront Surgery	Isotope + blue dye (control)	n = 137	94.1% (88.7–96.9%)	n = 33	0% (0–10.4%)
	ICG + blue dye (study group)	n = 116	95.6% (90.3–98.1%)	n = 18	5% (0.9–23.6%)
	Only ICG	n = 117	100% (96.8–100%)	n = 6	Low cases for FNR
Post-NAST	Isotope + blue dye (control)	n = 51	88.2% (76.1–94.4%)	n = 30	10% (3.5–25.6%)
	ICG + blue dye (study group)	n = 57	91.2% (80.5–96.1%)	n = 20	10% (2.8–30.1%)
	ICG (single tracer)	n = 23	95.6% (79.0–99.2%)	NA	Low cases with ALND + ve for FNR

## Data Availability

Data are contained within the article and supplementary materials.

## Supplementary Materials

The following supporting information can be downloaded at: https://www.mdpi.com/article/10.3390/cancers18061042/s1, Supplementary Case 1/Video S1: Intraoperative Visualization of Sentinel Node Using ICG in a Post-lumpectomy Case: A Demonstration of High Yield and Surgical Precision; Supplementary Case 2/Video S2: Beacon-Guided Sentinel Node Biopsy in Large Ptotic Breast: Integration of ICG Fluorescence in Therapeutic Mammoplasty; Supplementary Case 3/Video S3: ICG-Guided Sentinel Node Biopsy in a Post-NACT BRCA1-Positive TNBC Patient Undergoing Bilateral Mastectomy with Reconstruction

## Abbreviations

ACOSOG	American College of Surgeons Oncology Group
ALND	Axillary Lymph Node Dissection
BCT	Breast Conservation Therapy
DCIS	Ductal Carcinoma In Situ
DFS	Disease-Free Survival
ESMO	European Society for Medical Oncology
FNR	False-Negative Rate
ICG	Indocyanine Green
IDC	Invasive Ductal Carcinoma
ILC	Invasive Lobular Carcinoma
IQR	Interquartile Range
IR	Identification Rate
LCIS	Lobular Carcinoma In Situ
LD	Latissimus Dorsi
LMICsand	Low- and Middle-Income Countries
LTAP	Lateral Thoracic Artery Perforator Flap
NACT	Neoadjuvant Chemotherapy
NAST	Neoadjuvant Systemic Therapy
NCCN	National Comprehensive Cancer Network
NSM	Nipple-Sparing Mastectomy
OS	Overall Survival
pCR	Pathological Complete Response
PRC	Perfusion-Related Complications
PROMs	Patient-Reported Outcome Measures
QALY	Quality-Adjusted Life Year
QoL	Quality of Life
RT	Radiation Therapy
SD	Standard Deviation
SLN	Sentinel Lymph Node
SLNB	Sentinel Lymph Node Biopsy
TNBC	Triple-Negative Breast Cancer
