# Supplementary material for: Evaluating the Use and Feasibility of Indocyanine Green (ICG) as a Beacon of Precision in Sentinel Node Biopsy for Breast Cancer from an Oncoplastic Practice in India
Source: Cancers (Basel). 2026 Mar 23;18(6):1042. doi: 10.3390/cancers18061042 (PMC13025566; doi:10.3390/cancers18061042)
Supplement: Supplementary file 1 [file cancers-18-01042-s001.zip › cancers-4162506-non-published.pdf]

Evaluating the Use and Feasibility of Indocyanine Green (ICG) as a Beacon of Precision in Sentinel Node Biopsy for Breast Cancer from an Oncoplastic Practice in India

- Koppiker et. al. 2026

Videos Uploaded on the link:
https://doi.org/10.5281/zenodo.17745489
