# Supplementary material for: Evaluating the Use and Feasibility of Indocyanine Green (ICG) as a Beacon of Precision in Sentinel Node Biopsy for Breast Cancer from an Oncoplastic Practice in India
Source: Cancers (Basel). 2026 Mar 23;18(6):1042. doi: 10.3390/cancers18061042 (PMC13025566; doi:10.3390/cancers18061042)
Supplement: Supplementary file 1 [file cancers-18-01042-s001.zip › cancers-4162506-supplementary.pdf]

Supp Case:1 **Intraoperative Visualization of Sentinel Node Using ICG in a Post-lumpectomy Case: A Demonstration of High Yield and Surgical Precision**

Supp Figure 1 Case 1

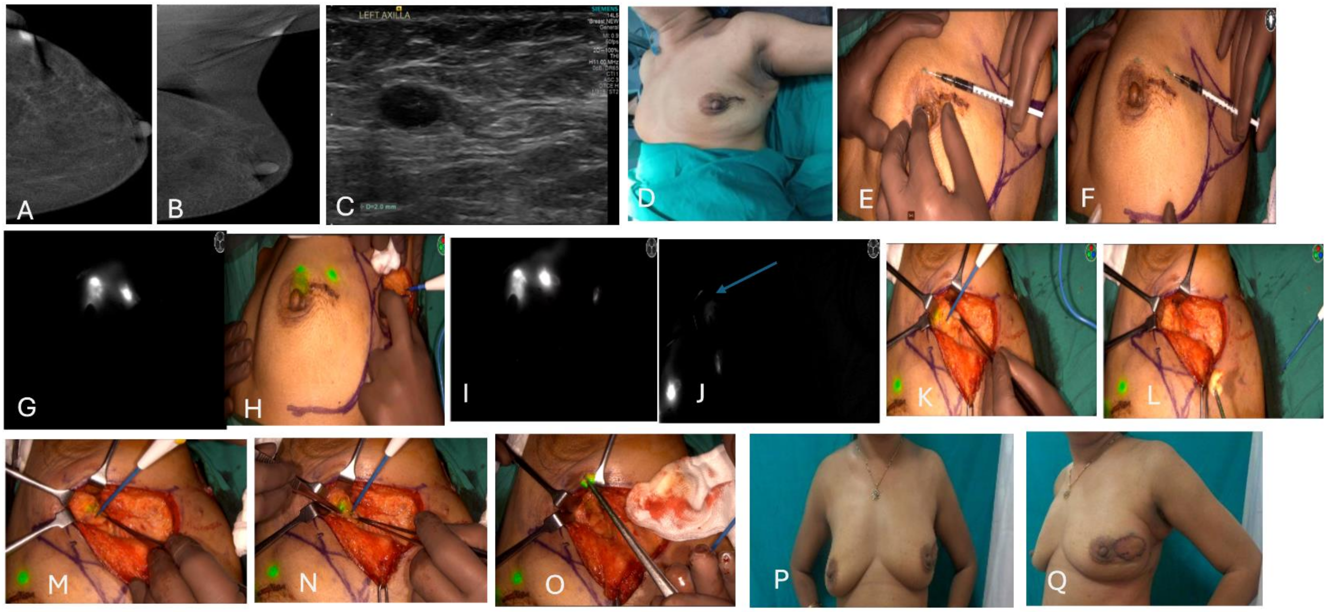


A,B,C) Radiology; D) Pre op; E,F) ICG injection; G) ICG pick up seen; H) Axillary incision; I,J) Sentinel Node lightening up; K,L) 1st Sentinel node pick up; M,N,O) 2nd Sentinel node pick up; P,Q) Post Op

Case Summary: This case illustrates a representative example of high-fidelity sentinel lymph node biopsy (SLNB) using indocyanine green (ICG) in a straightforward axillary dissection scenario, as captured in the accompanying surgical video.

A 47-year-old postmenopausal woman with medium-grade ptosis presented for further management after undergoing a lumpectomy at another center. Her histopathology report revealed a 1.9 × 1.3 cm IDC, grade III, with ER and PR positivity and HER2 negativity. Axillary nodal status was not reported.

A contrast-enhanced mammogram showed non-mass enhancement in the upper outer quadrant of the right breast, consistent with postoperative changes. No abnormal enhancement was noted in either breast, and no suspicious axillary lymphadenopathy was seen.

The patient was planned for a wide local excision of the lumpectomy cavity with volume replacement using a lateral intercostal artery perforator (LICAP) flap with skin paddle, along with ICG-guided sentinel lymph node biopsy (SLNB) of the left axilla.

Surgery was performed with the patient in a supine position. Preoperative markings were completed, and ICG was injected superolateral to the previous lumpectomy scar. Axillary access was achieved through a separate incision, and sentinel lymph nodes were visualized using black and white mode on the near-infrared imaging system. Two sentinel nodes were identified, excised, and sent for intraoperative frozen section analysis, both of which were negative for metastasis. High fluorescence intensity was confirmed on both overlay and color modes during node retrieval.

Margins of the excised cavity were adequately shaved and confirmed negative; no residual tumor was observed. The LICAP flap was raised and rotated into the cavity for volume replacement. Haemostasis was secured, a drain placed, and wound closure performed.

Final histopathology confirmed the original tumor size of 1.9 × 1.3 cm (pT), with clear margins and 0/2 sentinel nodes involved (pTx N0 Mx). The patient had an uneventful postoperative course.

Ref: Supp Video 1

Supp Case:2 **Beacon-Guided Sentinel Node Biopsy in Large Ptotic Breast: Integration of ICG Fluorescence in Therapeutic Mammoplasty**

Supp Figure 2 Case 2

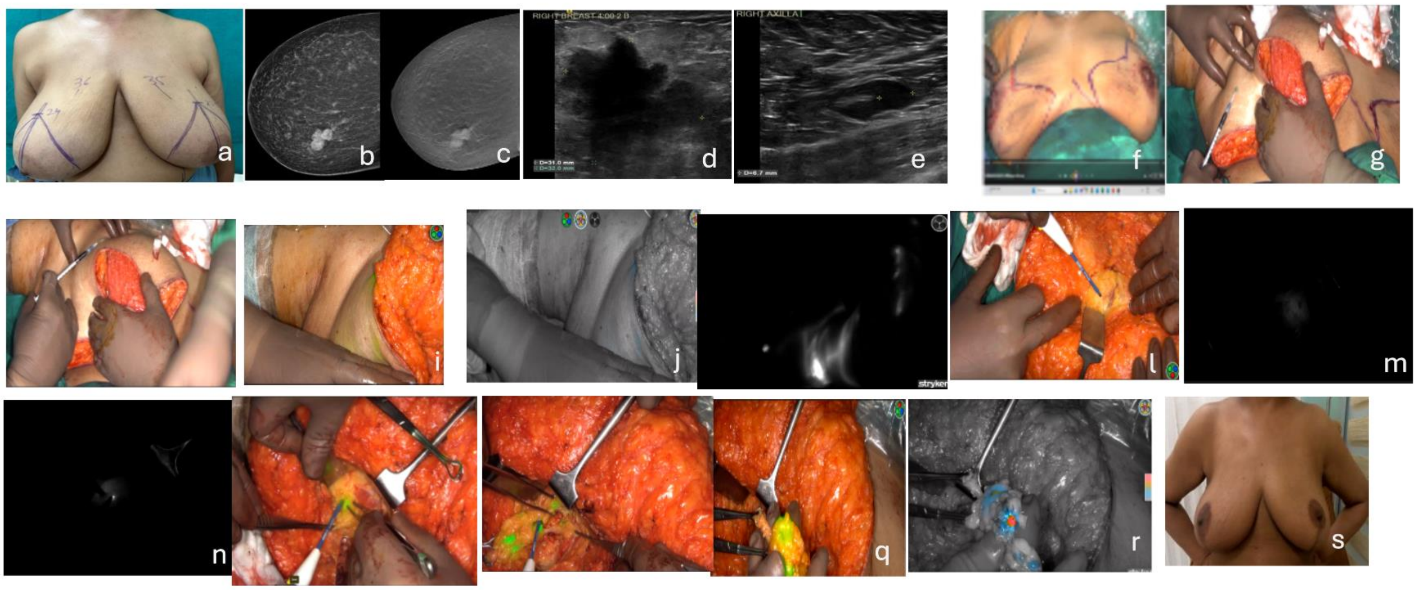

a) Pre Op; b, c, d) Lesion location on radiology; e) Axilla USG; f) Wise pattern incision; g, h) ICG dye injection; i,j,k) Dye perfusion; l) Sentinel node dissection; m, n) Beacon; o,p,q,r) Sentinel node pick up; s) Post Op

Case Summary: A 61-year-old postmenopausal woman with large, ptotic breasts presented with a palpable lump in the right breast. Diagnostic mammogram and targeted ultrasound revealed an irregular, dense mass with spiculated margins in the lower inner quadrant, corresponding to a lesion at 4 o'clock, measuring 31 × 32 mm. A duct-like extension measuring 7.3 cm superolateral to the index lesion raised suspicion of a DCIS component. No abnormal axillary nodes were noted. Trucut biopsy confirmed invasive carcinoma of no special type (NST), grade 2. Immunohistochemistry showed ER+/PR+/HER2– profile.

Given her large breast volume and potential complications of radiation therapy, she was advised to undergo wide local excision with therapeutic mammoplasty, ICG-guided sentinel lymph node biopsy (SLNB), and contralateral symmetrisation.

Under general anaesthesia, the patient was positioned supine, with standard sterile preparation and Wise-pattern markings. The tumour was localized intraoperatively using ultrasound and excised with surrounding tissue; the specimen was sent for frozen section. Margins were adequately shaved. ICG was injected into the lateral mammoplasty flap. The axilla was accessed, and under near-infrared guidance, the **"Beacon Sign"**—a focused flash of fluorescence—was visualized in black-and-white mode, guiding the dissection precisely to the sentinel nodes. High SLNB yield was confirmed using overlay and colour modes. Four sentinel lymph nodes were retrieved; two were positive on frozen section. Axillary closure was performed over a drain.

The nipple-areola complex was repositioned using a superomedial pedicle, and its vascularity was assessed intraoperatively with ICG, confirming good perfusion. Haemostasis was achieved and the wound closed.

Final histopathology revealed a 3.2 cm main tumour with a separate 4 mm lesion 1 cm away from the index tumour. Sentinel lymph node pathology showed 2 out of 4 nodes positive, consistent with pT2N1aMx. The patient received adjuvant chemotherapy and radiotherapy as per protocol.

**Note:** A supplementary surgical video from this case demonstrates real-time identification of sentinel nodes using the **Beacon Sign**, showcasing the precision and utility of ICG in oncoplastic surgery.

Ref: Supp Video 2

Supp Case 3**: ICG-Guided Sentinel Node Biopsy in a Post-NACT BRCA1-Positive TNBC Patient Undergoing Bilateral Mastectomy with Reconstruction**

Supp Figure 3 Case 3

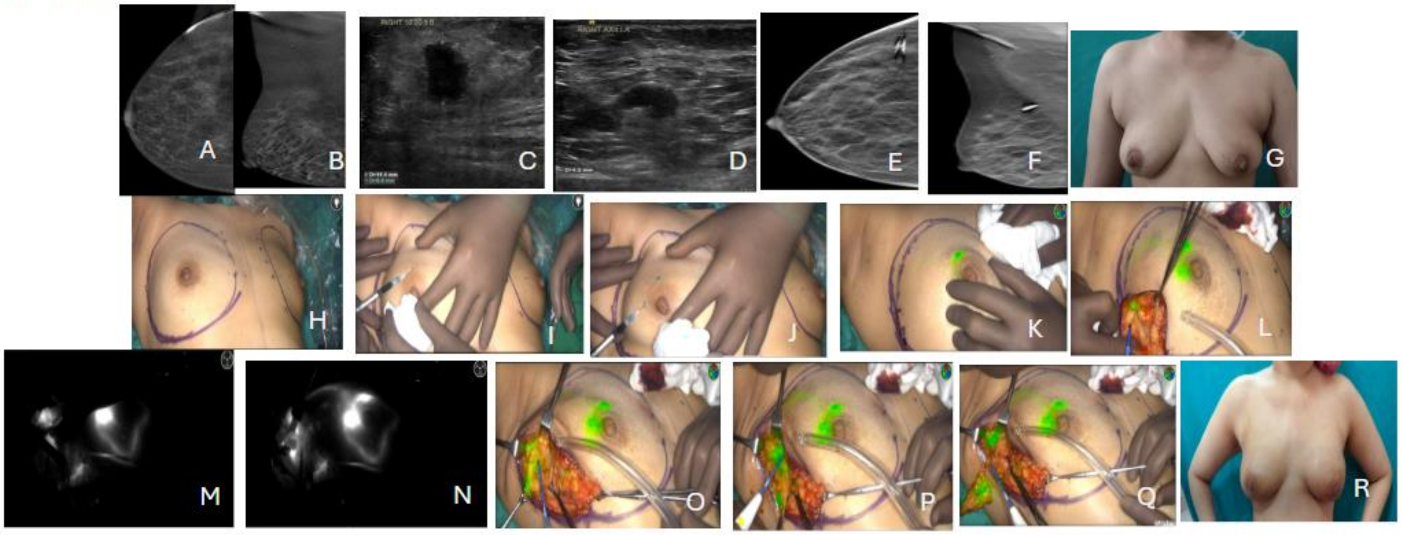


A,B,C,D) Pre NACt Radiology; E,F) Post NACt Radiology; G,H) Pre Op; I,J,K) ICG dye injected L) Lateral incision; M,N) Nodes seen; O,P,Q,) Sentinel node pick up; R) Post Op

Case Summary: A 44-year-old premenopausal woman with a strong family history of breast cancer presented with a lump in the right breast. Baseline contrast-enhanced spectral mammography revealed a heterogeneous non-mass enhancement in the upper outer quadrant measuring 7.7 × 4.5 cm and an irregular lesion at 10 o'clock, 3B region (11.4 × 8.6 mm). Two axillary lymph nodes with cortical thickness of 4.5 mm were noted. Trucut biopsy confirmed invasive carcinoma of no special type (NST), grade III. Immunohistochemistry revealed triple-negative receptor status (ER-, PR-, HER2-), and germline testing confirmed a BRCA1 mutation.

She received four cycles of neoadjuvant chemotherapy (NACT), following which radiologic reassessment showed no residual dominant mass or abnormal enhancement near the marker clip, suggesting good radiologic response.

Given her BRCA1 positivity, the patient opted for a right nipple-areola complex (NAC)-sparing mastectomy with latissimus dorsi (LD) flap reconstruction and SLNB, along with contralateral prophylactic mastectomy and prepectoral implant-based reconstruction.

Intraoperatively, ICG was injected in the periareolar region. A lateral incision on the right breast provided axillary access. Lymphatic channels were clearly visualized on near-infrared imaging. Four sentinel lymph nodes were identified using overlay and color-segmented modes (demonstrating high ICG uptake) and excised with precision using real-time fluorescence guidance. All nodes were negative on frozen section. The same incision was used to complete the right NAC-sparing mastectomy.

The patient was then repositioned laterally, and the LD flap was raised and rotated into the defect. Vascularity of the flap was confirmed using ICG. The left mastectomy was performed via an inframammary

incision and reconstructed with a prepectoral silicone implant. Haemostasis was achieved, and wounds were closed.

Final histopathology confirmed pathological complete response (ypT0N0Mx) with no residual tumor and 0/4 sentinel nodes involved. The patient has been on regular follow-up every three months and has since undergone prophylactic bilateral salpingo-oophorectomy.

This case highlights the feasibility of ICG-guided SLNB post-NACT, even in high-risk genetic mutation carriers undergoing complex bilateral reconstructive procedures. The high SLNB yield, real-time guidance, and accuracy demonstrate the practicality of ICG as a standalone tracer in post-NACT workflows.

Ref: Supp Video 3
